# Medical Topography of York Township, Michigan

**Published:** 1851-03

**Authors:** W. W. Goff

**Affiliations:** Moorville, Mich.


### ARTICLE II.

*Medical Topography of York Township, Washtenaw Co., Mich.*
By Dr. W. W. Goff, of Moorville, Mich.

The readers of etiological history, should not expect anything of the marvelous in the description of this quiet Township. Nothing here excites veneration or sublimity, except the solemn forest, which keeps its steady place; no towering mountains, no granite hills, no foaming cataract, no "silvery lake," no Mississippi floats through with its ocean of waters; nor is there so much as a Popocatapetl, to manufacture saltpeter or brimstone for us.

This township is bounded North by Pittsfield, East by Augusta, South by Milan, West by Saline. The population is about 1000, occupied principally in agriculture. Some parts of the township have been settled more than twenty years; but most of the settlement is of later date the emigration is principally from the State of New York.

*Rivers.* The Saline is a small stream of two or three feet water, at low water mark, running south east through the township. Its course is crooked, it has a feeble current and occasionally affords site to a mill; the water is colored and flavored more or less by the accumulations of floodwood at various places in its course, as well as by the constant supplies of drift, as leaves &c., from the burdening forest, reminding one of swamps, springs, &c. This stream like others in this part of the State, is subject to great variation in the wet and dry seasons, at times making pretensions to rank among rivers; then again making itself principally useful to mills, fish, and ——— geese. The Saline has a clay-like, miry bed, and low banks, which it frequently overflows.

The *soil* of this township is clay, clay and sand, and sand, clay predominates.

The *surface* is level in the south and east; more undulating in the north and west, but never rises to hills of much altitude, nor degenerates to swamps, except that it is occasionally springy, or boggy on the openings. The level lands require draining to make them profitable and arable, but are naturally good grass lands.

*Timber.* The clay soil, or clay and sand, is found mostly in the eastern and southern sections. These parts are heavily timbered with oak, ash, bass-wood, maple, hickory, black-walnut, shagbark, butternut, elm and beech, which attain respectable dimensions, and many of them formidable to the inexperienced woodsman. The northern and western parts of the township are called openings. The timber there is less varied and less abundant, a medium oak constituting the prevailing variety.

*Stone.* We have no quarries of stone here, nor do I know of any within twenty miles. The stone mostly used in walls, are found on the surface, irregular shaped, commonly called "hard heads," "sand stone," &c., almost defying a stone hammer to make them face two ways at the same time. These stone abound in some localities; whether they indicate regular stratified deposits adjacent, I am not aware, such have not yet been developed.

The *Medical Botany* of this township is the most considerable of its characteristics. Sanguinaria Canadensis, Geranium Maculutum, Cornus Florida, Asclepias Tuberosa, Prunus Virginiana, Eup. Perf. and the whole armature of the domestic and pepper treatment, are found here in profusion.

Here, as in the western country generally, *the* cause of disease is *Malaria*, or what is understood by that term, in some of its manifestations or degrees. Whether this is an existent acting principle in the causation of disease, or has its being only in the imagination of the wise ones, it answers well as a pack horse to bear the execrations of shivering humanity in this western country. There is no fountain heads of that poison, that I know of in this vicinity, except that the mill ponds, and streams should be so considered. The dam across the Saline near here, raises a pond for a mile, twice that by the stream; during the summer months this pond is kept full, or as near as possible.

My own experience is, that, other things being equal, there is no more disease on the course of the stream, or in the immediate vicinity of this pond, than in other partsof the township; nor that disease has different characteristics in these vicinities compared with other places. But there is a small stream four miles east, running nearly south, that has in some places a gravelly bed, that is damed for the support of a saw mill; this dam courses a large marshy pond, which is damned by all the inhabitants in its vicinity, as one of the greatest morbific inflictions ever imposed upon a neighborhood. Dr. Bowers, a physician for many years a resident here, informs me, that the pond first mentioned, adds materially to the amount of disease in this locality. The dam was down and the river had unobstructed course for six years, 1839 to 1845; during this period there was much less disease than anterior or subsequently, since the first erection of the dam in 1834.

The prevalent diseases are intermitting and remitting fevers, bilious pneumonia, diarrhoea dysentery, and occasionally rheumatism, and some of the inflammations; these are *prevalent*, but additional, we have more or less of the whole nosological list under care at one time or another, but nearly all diseases are modified by the ever present genius of evil—malaria.

My professional experience in this place dates only one year. The epidemic diseases of the past season, 1850, showed the usual characteristics of malarious origin, generally. During the period, the month of September particularly, when diseases took definite character, there was a diarrhoæ for two, four or six days of a chylous or *a bilious* character; discharges liquid, light colored; affording temporary relief to the patient; no complaint of tormina nor tenesmus in most cases; patient uniformly expecting that some mild astringent would affect a cure. Many of these cases were attended with a mild quotidian or tertian. As the disease became developed, the patient had nausea and vomiting more or less, alternating, and diarrhoea; great oppression of the precordial region, general uneasiness, amounting to pain in many instances, of the portal region; the intermittent or remittent assumed a severe form, well developed, the chill and fever occupying 12 to 20, in some cases 48 hours. After the second or third paroxysm, unless molested by treatment, assuming more of a continued character, the remission not being perceptible, or, very indistinct, and congestions formed.

In the forming stage, the treatment found the most successful, was to exhibit Calomel and Jalap, a a gr. 8 or 10, or 8 to 12 gr. cal., followed by castor oil, jalap, or jalap and cr. tartar, in 2 to 6 drs. In many cases, this treatment, with one to three five grain doses of Quinine to check the intermittent, supported by some of the bitter tonics, removed the malady.

But too often the disease established itself, then calomel in limited quantities, laxatives, febrifuges, as camphor powders, (℞ camph. carb. am. a a 1ʒ Ipecacʒij( Dovers powders, or if there was much heat and restlessness, cold water, varied to meet the exigencies of particular cases, established convalescence; and the infusions of Gentian, Orange perl., quassia, or wild cherry, with perhaps two or three grs. sul. iron in the infusion, generally concluded the treatment.

